# Platelet-rich plasma use in meniscus repair treatment: a systematic review and meta-analysis of clinical studies

**DOI:** 10.1186/s13018-022-03293-0

**Published:** 2022-10-08

**Authors:** Ziquan Li, Xisheng Weng

**Affiliations:** 1 Department of Orthopedic Surgery, Peking Union Medical College and Chinese Academy of Medical Sciences, Peking Union Medical College Hospital, No. 1 Shuaifuyuan, Beijing, 100730 China; 2 State Key Laboratory of Complex Severe and Rare Diseases, Peking Union Medical College and Chinese Academy of Medical Sciences, Peking Union Medical College Hospital, Beijing, 100730 China

**Keywords:** Meniscus repair, Platelet-rich plasma, Platelet-rich fibrin matrix, Meta-analysis, Systematic review, Subgroup analysis

## Abstract

**Background:**

There is conflicting clinical evidence whether platelet-rich plasma (PRP) therapies could translate to an increased meniscus healing rate and improved functional outcomes. The objective of this systematic review and meta-analysis was to compare the failure rate and patient-reported functional outcomes in meniscus repair augmented with and without PRP.

**Methods:**

We comprehensively searched the PubMed, Web of Science, Medline, Embase, and Cochrane Library databases to identify studies that compared the clinical efficacy of meniscus repair performed with PRP versus without PRP. The primary outcome was the meniscus repair failure rate, while the secondary outcomes were knee-specific patient-reported outcomes, including the International Knee Documentation Committee (IKDC) score, Lysholm knee scale, visual analog scale, Tegner activity level score, Western Ontario and McMaster Universities Osteoarthritis Index score, Single Assessment Numeric Evaluation score, and Knee injury and Osteoarthritis Outcome Score. Furthermore, subgroup analyses were performed by stratifying the studies according to the PRP preparation technique to investigate the potential sources of heterogeneity among studies.

**Results:**

Our meta-analysis included nine studies (two RCTs and seven non-RCTs) with 1164 participants. The failure rate in the PRP group was significantly lower than that in the non-PRP group [odds ratio: 0.64, 95% confidence interval (CI) (0.42, 0.96), *P* = 0.03]. Furthermore, the PRP group was associated with a statistically significant improvement in the visual analog scale for pain [Mean difference (MD): − 0.76, 95% CI (− 1.32, − 0.21), *P* = 0.007] and Knee injury and Osteoarthritis Outcome Score-symptom [MD: 8.02, 95% CI (2.99, 13.05), *P* = 0.002] compared with the non-PRP group. However, neither the IKDC score nor the Lysholm knee scale showed any differences between the two groups. In addition, the results of subgroup analyses favored PRP over platelet-rich fibrin matrix (PRFM) regarding the IKDC score.

**Conclusions:**

Although meniscus repairs augmented with PRP led to significantly lower failure rates and better postoperative pain control compared with those of the non-PRP group, there is insufficient RCT evidence to support PRP augmentation of meniscus repair improving functional outcomes. Moreover, PRP could be recommended in meniscus repair augmentation compared with PRFM. PRFM was shown to have no benefit in improving functional outcomes.

## Introduction

The menisci are fibrocartilaginous structures in the tibiofemoral joint. A complete meniscus structure has the functions of lubrication, nutrition, joint stability, shock absorption, and load transmission during dynamic movements [1, 2]. Meniscus injury is thought to be a unique challenge because of the absence of healing at the avascular zone, the instability of the knee joint, the accelerated degeneration of articular cartilage, and the increased rate of early-onset osteoarthritis [3, 4]. At present, it is the consensus that meniscus repair or stimulation of meniscus regeneration in the treatment of meniscus injury could potentially prevent or delay osteoarthritis onset [5]. However, the improvement of regeneration and the increase in the healing rate after injury have been significant challenges.

Platelet-rich plasma (PRP) is defined as an autologous blood-derived product that contains highly concentrated platelets, associated growth factors, and other bioactive components [6]. PRP has been demonstrated to display positive effects on tissue healing by stimulating cell proliferation, cell migration, angiogenesis, and extracellular matrix production in numerous cell types in both in vitro and in vivo models [7, 8]. Despite a paucity of large-scale clinical evidence to support the use of PRP therapy, there has been widespread application for various musculoskeletal injuries involving tendon, ligament, cartilage, and/or bone owing to the enthusiasm regarding its potential [9–12]. Many PRP growth factors, including platelet-derived growth factor and transforming growth factor beta, have been shown to modulate the inflammatory process and regulate chondrocyte viability, contributing to tissue maintenance and meniscus repair [13–15]. Furthermore, various clinical studies have verified that PRP injection provided good functional scores and radiological improvement in the patients with symptomatic meniscal lesions [1, 16–18]. By contrast, several retrospective comparative studies showed that there were no significant improvements in pain relief or functional improvement on PRP application in meniscus repair [19–21].

Consequently, the effectiveness of PRP for meniscus repair is greatly debated. Although there have been three published systematic review studies on PRP augmentation in meniscus repair treatment [22–24], their major limitations were that they included only a few studies with a limited number of patients as well as studies with heterogeneity of different PRP preparations. Therefore, the aim of present investigation was to systematically review and perform a meta-analysis of the literature to investigate PRP efficacy in meniscus repair, including the most recent matched case–control studies [25, 26]. In addition, different forms of PRP preparations were evaluated by subgroup analyses.

## Methods

### Literature search and selection criteria

Our systematic review and meta-analysis were performed in the PROSPERO registration (No. 300489). The Preferred Reporting Items for Systematic Reviews and Meta-Analyses (PRISMA) checklist was used. Two independent reviewers performed the literature search in accordance with the PRISMA guidelines and reviewed the search results. The PubMed, Web of Science, Medline, Embase, and the Cochrane Library databases were systematically searched. No publication date restriction was applied. To include all the articles about the clinical efficacy of meniscus repair performed with PRP, a structured literature search was applied using the following string: ((“PRP” OR “platelet-rich plasma” OR “plasma-rich fibrin”) AND (“meniscus” OR “menisci” OR “meniscal”)).

The adopted inclusion criteria were as follows: (1) original articles; (2) comparative studies involving meniscus repair with or without PRP augmentation; (3) the studies were analyzed with at least one of the following outcomes: visual analog scale (VAS) scores, meniscus repair failures and knee-specific patient-reported outcome scores; and (4) full-text articles available in English. Exclusion criteria were as follows: (1) studies including patients undergoing other surgical treatments unrelated to meniscus repair; (2) PRP not the sole difference between the experimental and the control group; (3) animal studies, basic science investigations, review articles, or technique papers; and (4) articles published in other languages. We retrieved the full texts of eligible studies, and only the most recent or the single article provided with the most information was included when duplicates were identified.

### Data extraction and quality assessment

The following data from studies were extracted by two independent reviewers: the first author’s name, publication year, country, sample size, type and dosage of PRP implementation, follow-up time, and the characteristics of the study population. Clinical outcomes recorded were the meniscus repair failure rate and knee-specific patient-reported outcomes: International Knee Documentation Committee (IKDC) score, Lysholm knee scale, VAS scores for pain, Tegner activity level score, Western Ontario and McMaster Universities Osteoarthritis Index (WOMAC) score, Single Assessment Numeric Evaluation (SANE) score, and Knee injury and Osteoarthritis Outcome Score (KOOS). PRP preparations were classified into four subtypes: leukocyte-poor (LP) pure PRP, leukocyte-rich (LR) pure PRP, LP platelet-rich fibrin matrix (PRFM), and LR PRFM [27]. Studies were defined as LP/LR or PRP/PRFM by the manufacturers’ specifications and whether they had more or fewer leukocytes than autologous blood. Where requisite data were lacking in the publications, the original investigators were contacted.

The Newcastle-Ottawa Quality Assessment Scale (NOS) was used for assessing non-randomized studies with the following three broad categories: selection (S1: Definition of cases; S2: Representativeness of the cases; S3: Selection of controls; S4: Adequate control definition), comparability (C1: Comparability of cases; C2: Study controls for the basis of the analysis), and exposure (E1: Ascertainment of the exposure; E2: Ascertainment of the same method used for cases and controls; E3: Non-response rate) [28]. This scale is assigned from 0 to 9 points, with studies scoring below 6 points considered low quality, 6 and 7 points represent moderate quality, while 8 and 9 points indicate high quality [29]. The quality of randomized controlled trials (RCTs) was assessed by the criteria outlined in the Cochrane Handbook for Systematic Reviews of Interventions, including the following items: random sequence generation, allocation concealment, blinding of participants and personnel, blinding of outcome assessment, incomplete outcome data, selective outcome reporting, and other bias.

### Statistical analysis

Meta-analysis was conducted in Review Manager (RevMan Version 5.4, The Cochrane Collaboration, Oxford, UK) and STATA version 14.0 (Stata Corporation; College Station, TX, USA). Continuous outcomes were addressed as standardized mean differences (MDs), and the dichotomous data were expressed as odds ratios (ORs). The effect sizes were reported with 95% confidence intervals (95% CIs). Heterogeneity of an article was estimated by the chi-squared test and *I*^2^ statistic. If in the chi-squared test *P* < 0.1 or the *I*^2^ statistic > 50%, heterogeneity was considered to be significant, and a random effect model was used to decrease the impact of heterogeneity on the results in this situation. Otherwise, a fixed-effects model was applied for the meta-analysis. Sensitivity analyses and subgroup analyses were conducted to investigate possible sources of heterogeneity and the stability of the results. *P* values of < 0.05 were considered to be statistically significant.

## Results

### Study selection

**Fig. 1:**
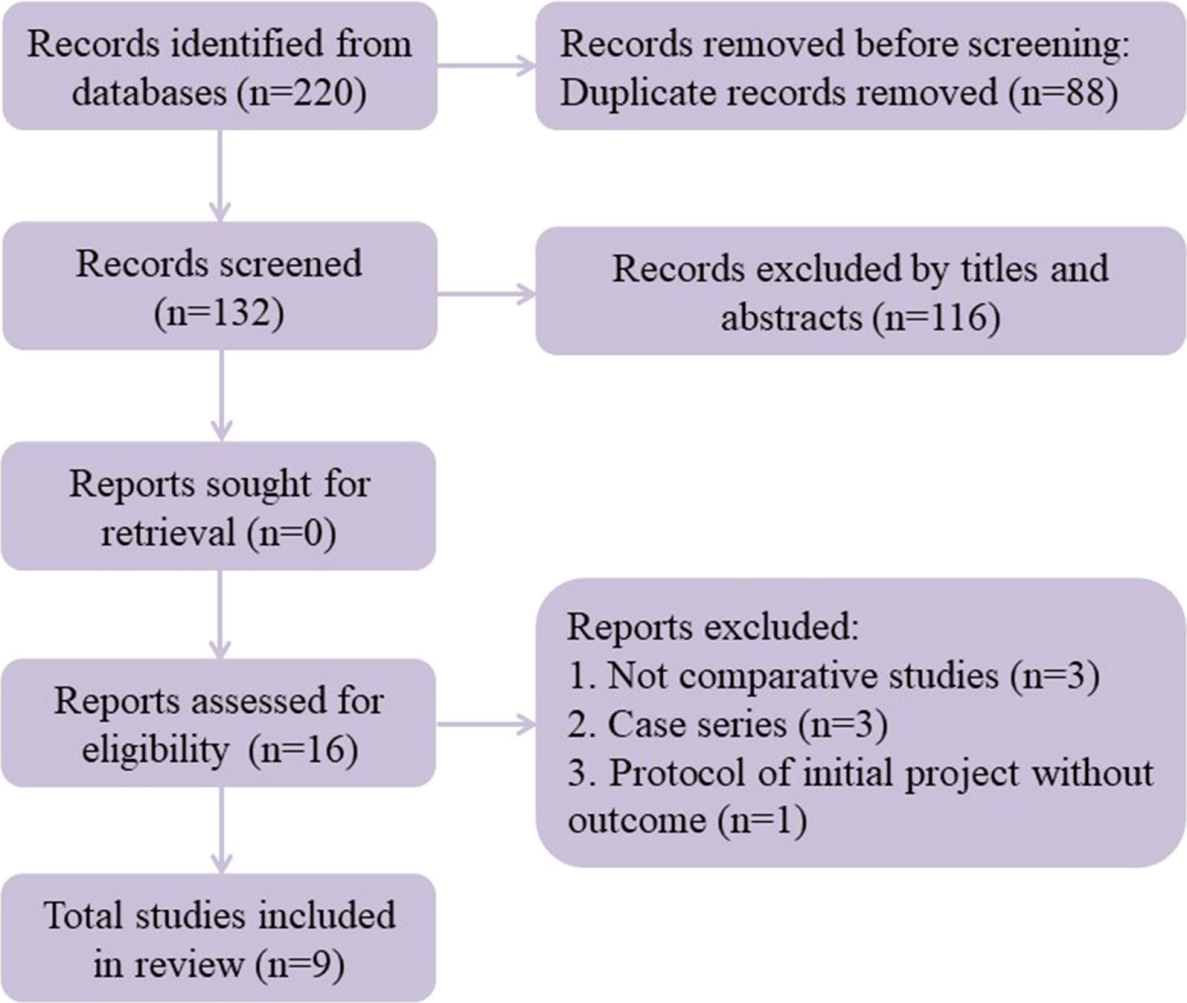
Flow diagram of the meta-analysis

Our search strategy initially identified 220 possible studies, and no additional records were found during manual searches of references. After removing 88 duplicate studies, a total of 116 records were excluded because they were deemed irrelevant according to the title and abstract. After thoroughly reviewing the full texts of the 16 potentially eligible articles, nine articles (two RCTs and seven non-RCTs) were selected for the final analysis, which were published between 2015 and 2021 [19, 20, 25, 26, 30–34]. A flowchart depicting the study selection strategy is shown in Fig. 1.

### Study characteristics and quality assessment

Nine articles with 1164 participants were included in this systematic review: 519 augmented with PRP (experimental group) and 645 without PRP (control group). All participants were diagnosed with meniscal injuries based on physical examination, magnetic resonance imaging (MRI), or arthroscopy at the time of surgery. In addition, the treatment options and PRP preparation and administration varied among the enrolled studies. We identified six studies in which PRP was delivered arthroscopically and sutured or injected into the repair site [19, 20, 25, 31–33], one study with in situ PRP injection into the repaired lesion after mini-arthrotomy [30], one study with multiple intra-articular PRP injections after meniscus repair surgery [26], and one study with minimally invasive (percutaneous) intrameniscal PRP application [34]. Furthermore, there were seven studies using LR-PRP, with two other studies not specifying LR versus LP formulations [20, 26]. Two studies utilized PRP with a fibrin matrix [20] or clot [32] that was inserted into the repair site. The remaining seven studies injected thrombin-activated PRP into the meniscus repair site. Detailed information about the baseline and characteristics are presented in Table 1.

**Table 1:** Demographic data of the included studies

References	Country	Design/level of evidence	Sample size (PRP/control)	BMI	Mean age (years)	Gender (M/F)	PRP preparation	PRP injection protocol	PRP/PRFM	LR/LP	Follow-up (months)
Yang et al. [26]	China	Retrospective comparative trial/III	61 (30/31)	25.2	36.4	44/17	Regen Kit	Multiple intra-articular injection after surgery	PRP	NR	33
Bailey et al. [25]	America	Retrospective matched case–control study/III	324 (162/162)	25.6	22.2	192/132	Angel double-spin, concentrated PRP system	Intraoperative administration	PRP	LR	24
Kaminski et al. [34]	Poland	Prospective, randomized, placebo-controlled, double-blind study/I	72 (42/30)	27.4	44.8	41/31	Activated using autologous thrombin	Minimally invasive intrameniscal application (6–8 mL)	PRP	LR	23
Everhart et al. [33]	America	Cohort study/III	550 (203/347)	27.6	28.8	348/202	GPS III system and Angel system	Introduced into the joint before closure (5 mL)	PRP	LR	36
Dai et al. [19]	China	Retrospective cohort/III	29 (14/15)	NR	31.3	11/18	Sodium citrate as anticoagulant	Injected on the repaired site under arthroscope (4 mL)	PRP	LR	20.6
Kemmochi et al. [32]	Japan	Prospective, interventional, non-randomized trial/II	22 (17/5)	NR	29.8	12/10	PRF box	Inserted into the cleft of the injured meniscus (2.4 cm^3^)	PRFM	LR	6
Kaminski et al. [31]	Poland	Prospective, randomized, double-blind, placebo-controlled, parallel-arm study/I	37 (19/18)	NR	28.1	30/7	ELISA and blood analyzer; Activated using autologous thrombin	Injected into meniscal repair site (8 mL)	PRP	LR	54
Pujol et al. [30]	France	Retrospective cohort/III	34 (17/17)	NR	30.3	24/10	GPS III system	In situ injection after mini-arthrotomy (5 mL)	PRP	LR	32.2
Griffin et al. [20]	America	Retrospective cohort /III	35 (15/20)	25	31.0	28/7	Cascade Platelet-Rich Fibrin Matrix	Sutured into meniscal repair site	PRFM	NR	48

*PRP* Platelet-rich plasma; *PRFM* Platelet-rich fibrin matrix; *LP* Leukocyte-poor; *LR* Leukocyte-rich; *BMI* Body mass index; *ELISA* Enzyme linked immunosorbent assay; *NR* Not report

Following the instructions in the Cochrane Handbook for Systematic Reviews of Interventions, five aspects related to the risk of bias were assessed in two RCTs [31, 34], including allocation, blinding, incomplete outcome data, selective reporting, and other potential sources of bias. In both these RCT studies, the patients, data collectors, and assessors were blinded and the random component in the sequence generation process was described. All participant exclusions and excessive drop-out in each study were reported. The results of the NOS for the quality of the seven non-RCTs are presented in Table 2. Five studies were graded as good [19, 25, 26, 30, 32] and two graded as fair [20, 33]. In particular, the studies by Griffin et al. and Everhart et al. [20, 33] were deemed problematic because they lacked the representativeness of the cases and did not take adequate actions to avoid bias in the study analysis.

**Table 2:** Risk of bias for non-randomized studies

	Selection	Comparability	Exposure	Assessment
Yang et al. [26]	Low (4 pts)	Low (2 pts)	High (2 pts)	Good
Bailey et al. [25]	Low (4 pts)	High (1 pt)	Low (3 pts)	Good
Everhart et al. [33]	Low (3 pts)	High (1 pt)	Low (3 pts)	Fair
Dai et al. [19]	Low (4 pts)	High (1 pt)	Low (3 pts)	Good
Kemmochi et al. [32]	Low (4 pts)	Low (2 pts)	High (2 pts)	Good
Pujol et al. [30]	Low (4 pts)	High (1 pt)	Low (3 pts)	Good
Griffin et al. [20]	Low (3 pts)	High (1 pt)	High (2 pts)	Fair

### Failure rate

**Fig. 2:**
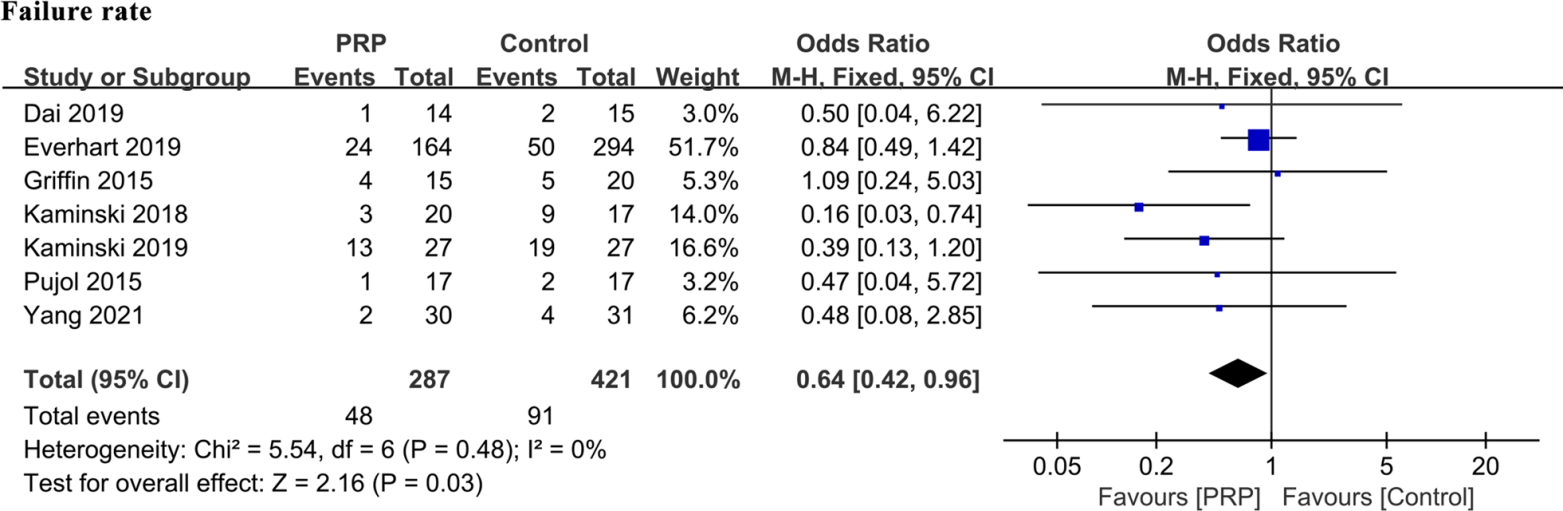
Comparisons of the failure rate between the PRP and control groups

A total of 708 patients from seven studies were considered to display repair failure when they developed a recurrence of meniscal symptoms and requirement for reoperation, or this was shown when evaluated by second-look arthroscopy or MRI postoperatively [19, 20, 26, 30, 31, 33, 34]. In the PRP group, 16.7% of patients experienced treatment failure, while in the control group, this was 21.6%. The result of the meta-analysis showed that the failure rate in the PRP group was significantly lower than that in the control group (OR: 0.64, 95% CI [0.42, 0.96], *P* = 0.03). There was low heterogeneity in the outcomes between the groups (*P* = 0.48, *I*^2^ = 0%) (Table 3, Fig. 2).

**Table 3:** Clinical outcomes of the included studies

Clinical outcomes		Yang et al. [26]	Bailey et al. [25]	Kaminski et al. [34]	Everhart et al. [33]	Dai et al. [19]	Kemmochi et al. [32]	Kaminski et al. [31]	Pujol et al. [30]	Griffin et al. [20]
IKDC score	PRP	75.1 ± 13.6	87.6 ± 13.3				87.4 ± 10.4	97.56 ± 0.63	90.7	69 ± 26
	Control	72.6 ± 15.8	88.1 ± 12.6				91.5 ± 1.2	84.77 ± 0.92	87.9	76 ± 17
	*P* value	0.593	0.952				0.13	0.001		0.288
Lysholm knee scale	PRP	80.6 ± 14.9				79.8 ± 9.6	95.8 ± 7.1			66 ± 31.9
	Control	77.7 ± 17.2				74.6 ± 11.6	97.2 ± 1.8			89 ± 9.7
	*P* value	0.670				0.306	0.69			0.065
VAS score	PRP			1.97 ± 0.05		1.2 ± 1.0		0.84 ± 0.10		
	Control			2.05 ± 0.08		1.6 ± 1.1		0.89 ± 0.08		
	*P* value			0.39		0.321		0.15		
SANE score	PRP		91.6 ± 11.2							
	Control		92.4 ± 10.6							
	*P* value		0.599							
WOMAC score	PRP			9.72 ± 0.32				0.95 ± 0.13		
	Control			7.50 ± 0.59				3.95 ± 0.33		
	*P* value			0.21				0.002		
Tegner activity level score	PRP						5.9 ± 2.3			
	Control						7.8 ± 1.6			
	*P* value						0.11			
Failure rate	PRP	6.7%		48.0%	14.6%	7.1%		15%	5.8%	26.7%
	Control	12.9%		70.0%	17.0%	13.3%		53%	11.8%	25.0%
	*P* value	0.874		0.04	0.60	0.58		0.048	0.54	0.89
*KOOS*										
(i) Pain	PRP			87.24 ± 0.36				96.06 ± 0.23	93.3	
	Control			89.00 ± 0.63				92.85 ± 0.43	78.4	
	*P* value			0.22				0.035	0.046	
(ii) Symptoms	PRP			92.03 ± 0.27				96.23 ± 0.31	90.9	
	Control			90.42 ± 0.56				92.33 ± 0.48	86.1	
	*P* value			0.27				0.029		
(iii) ADL	PRP			89.36 ± 0.36				98.18 ± 0.13	97.2	
	Control			92.38 ± 0.61				95.14 ± 0.38	93.8	
	*P* value			0.25				0.0004		
(iv) Sport/recreation	PRP			69.52 ± 0.77				89.44 ± 0.86	88.8	
	Control			78.98 ± 1.10				77.56 ± 1.26	74.4	
	*P* value			0.11				0.009	0.03	
(v) QoL	PRP			67.06 ± 0.55				80.90 ± 1.09	78.3	
	Control			68.18 ± 1.08				66.18 ± 1.17	74.6	
	*P* value			0.42				0.008		

*PRP* Platelet-rich plasma; *VAS* Visual analog scale; *IKDC* International knee documentation committee; *WOMAC* Western Ontario and McMaster universities Osteoarthritis index; *KOOS* Knee injury and osteoarthritis outcome score; *SANE* Single assessment numeric evaluation; *ADL* Activities of daily living; *QOL* quality of life. *P* value: Differences in continuous variables were assessed by two-tailed Mann–Whitney *U* test or unpaired *t* test and a chi-square test was used for categorical variables. A Kaplan–Meier survival plot was created for meniscal repair failure rate

### Patient-reported outcomes

**Fig. 3:**
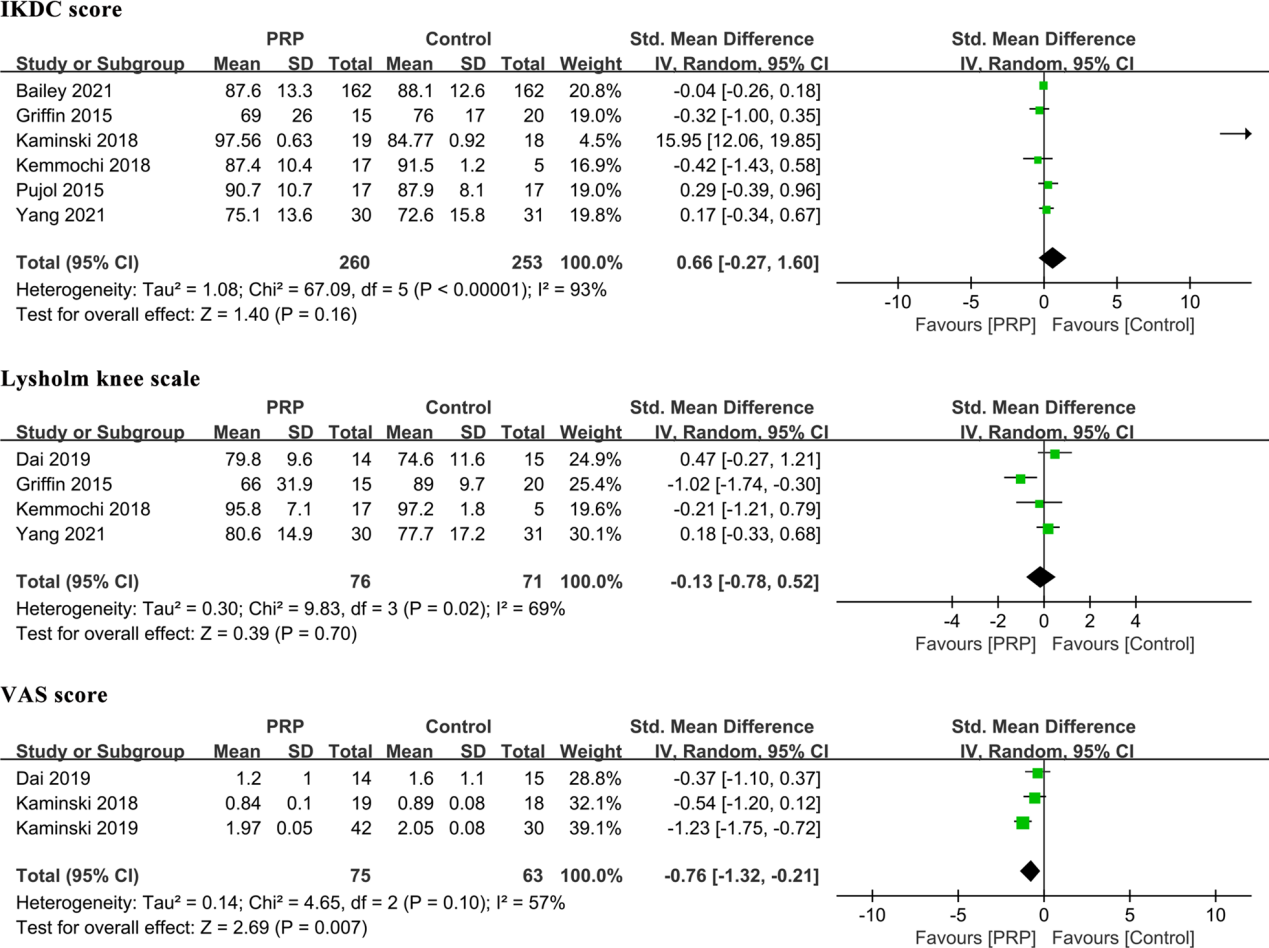
Comparisons of patient-reported outcomes between the PRP and control groups

The VAS score for pain was reported in three studies, with 75 patients treated with PRP and 63 with a control [19, 31, 34]. There was a statistically significant difference favoring PRP [MD: − 0.76, 95% CI (− 1.32, − 0.21), *P* = 0.007]. Random effects models were used because of statistical heterogeneity through meta-analysis (*P* = 0.10 and *I*^2^ = 57%). However, because the number of included studies was small, subgroup analyses were not performed (Table 3, Fig. 3).

The IKDC score was obtained from six studies involving 513 participants [20, 25, 26, 30–32]. The pooling results showed that the IKDC score was not significantly different between the PRP and control groups [MD: 0.66, 95% CI (0.27, 1.60), *P* = 0.16, *I*^2^ = 93%], and neither was the Lysholm knee scale [MD: − 0.13, 95% CI (− 0.78, 0.52), *P* = 0.70, *I*^2^ = 69%] (Table 3, Fig. 3). There was considerable heterogeneity among the studies. Sensitivity analysis did not detect the source of the heterogeneity, and we performed subgroup meta-analysis to investigate the possible sources of heterogeneity among the studies according to various confounding factors.

**Fig. 4:**
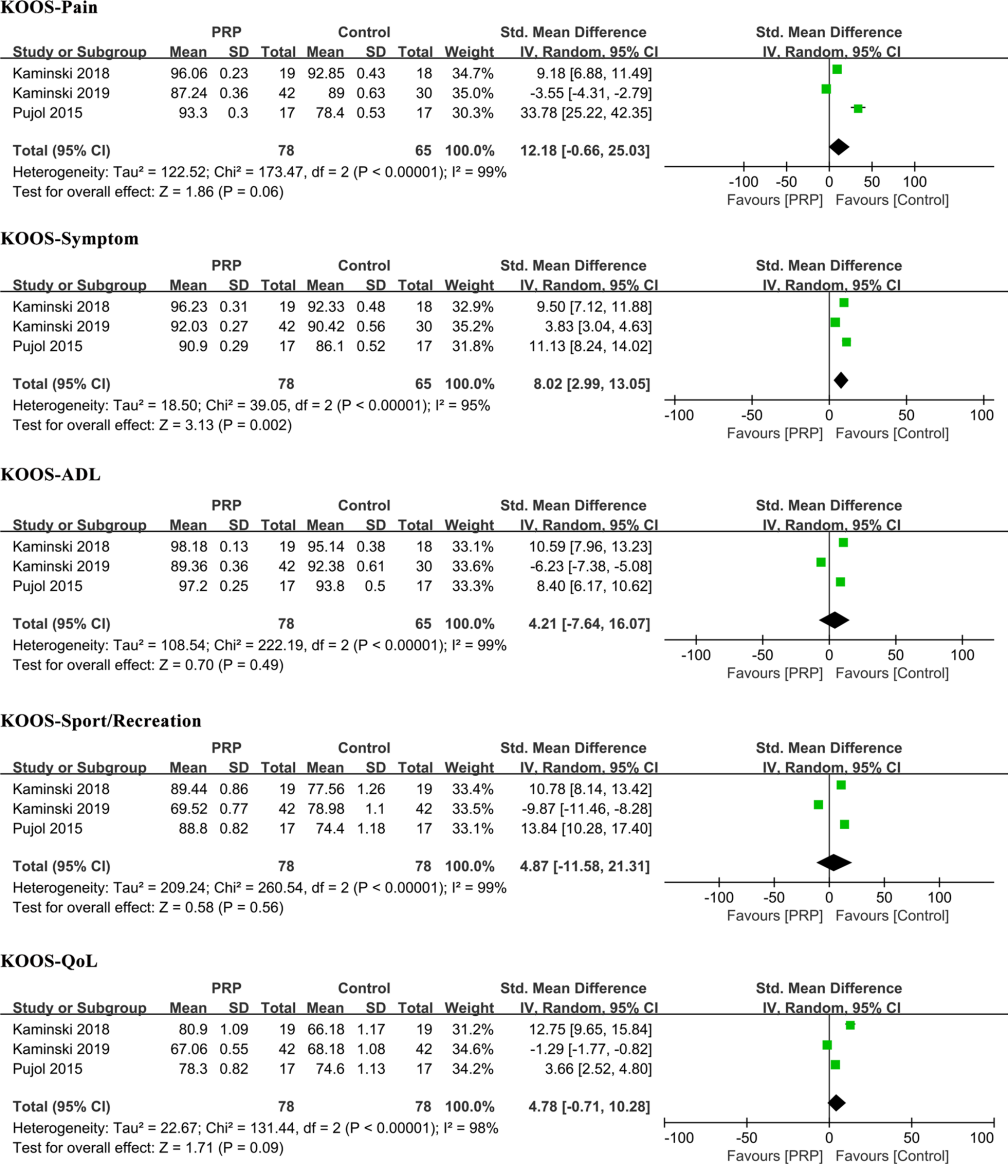
Comparisons of KOOS subscales between the PRP and control groups

All five KOOS subscales, pain, symptom, activity of daily living (ADL), sport/recreation, and quality of life (QoL), were recorded in three studies, with 78 patients treated with PRR and 65 with a control [30, 31, 34]. When the data of all studies were pooled, there was a significant difference between the PRP group and controls in KOOS-symptom [MD: 8.02, 95% CI (2.99, 13.05), *P* = 0.002, *I*^2^ = 95%], while no significant differences were detected between the two groups regarding KOOS-pain, KOOS-ADL, KOOS-sport/recreation or KOOS-QoL [MD: 12.18, 95% CI (− 0.66, 25.03), *P* = 0.06; MD: 4.21, 95% CI (− 7.64, 16.07), *P* = 0.49; MD: 4.87, 95% CI (− 11.58, 21.31), *P* = 0.56; and MD: 4.78 95% CI (− 0.71, 10.28), *P* = 0.09, respectively]. Sensitivity analysis failed to eliminate heterogeneity, and random effects models were adopted for analysis (Table 3, Fig. 4).

Furthermore, the Tegner activity level score [32] and SANE score [25] were each applied in one study, neither of which found significant differences in self-reported knee function scores between the PRP and matched-control groups. The subjective WOMAC score was used in two studies [31, 34]. Of these, one study found significantly better scores in the PRP group (*P* = 0.002) [31], however, the other study showed no statistical difference between the two groups (Table 3) [34].

### Subgroup analyses of the different varieties of PRP applications

Furthermore, subgroup analyses were performed to investigate the potential sources of heterogeneity among studies. This analysis was performed by stratifying the studies according to the PRP preparation technique. PRP and PRFM are both processed from autologous blood, but they differ in their preparation methods [35]. PRP is collected with anticoagulant and is immediately processed, whereas PRFM is collected immediately without anticoagulant, such that it forms a fibrin-rich clot that has to be sutured into the repair site or using a specialized delivery system.

**Fig. 5:**
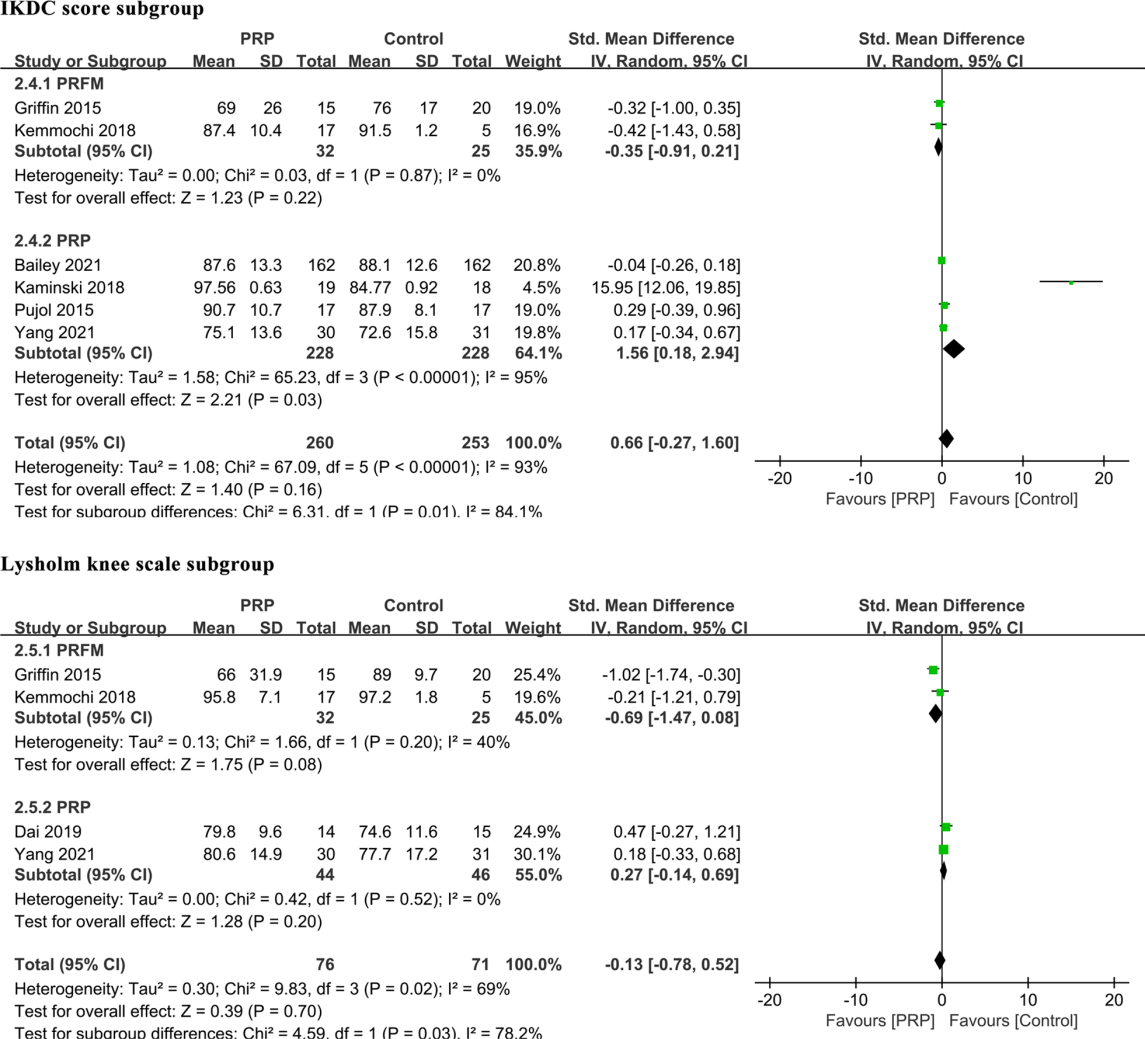
Subgroup analysis of the association between the different forms of PRP applications

Regarding the IKDC score, subgroup analysis showed that PRFM had no significant association with the IKDC score [MD: − 0.35, 95% CI (− 0.91, 0.21), *P* = 0.22]. However, a significant correlation between PRP and the IKDC score was observed [MD: 1.56, 95% CI (0.18, 2.94), *P* = 0.03]. In addition, we conducted subgroup analysis based upon the Lysholm knee scale. There was no significant association between the Lysholm knee scale and different preparation techniques of PRP [MD: 0.27, 95% CI (− 0.14, 0.69), *P* = 0.20] or PRFM [MD: − 0.69, 95% CI (− 1.47, 0.08), *P* = 0.08] (Fig. 5).

## Discussion

Injuries to the menisci are the most common condition among knee joint-related morbidities, can hardly heal, and frequently progress into structural breakdown, followed by the initiation of osteoarthritis [36]. With the importance of the meniscus in joint function and diseases becoming recognized, there is a need for an accelerated and performant healing strategy [37]. Biologic augmentation techniques, including PRP, have gained significant interest as viable treatment options to enhance repair healing following meniscus injury [23]. Some basic science and clinical studies have suggested that PRP is gaining increasing attention as an adjuvant for meniscus repair and its safety has been established, whereas other studies have shown the opposite regarding the repair [25, 38]. Therefore, in the present systematic review and meta-analysis, which was based on nine studies with 1164 participants, the results showed a trend that PRP augmentation could reduce the repair failure rate and provide better postoperative pain control. However, there were no significant differences with regard to most of the patient-reported functional outcomes. The results of subgroup analyses favored PRP over PRFM regarding the improvement of functional outcomes.

The beneficial effect of PRP on the meniscus repair failure rate is supported by prior basic science research, because an in vitro and animal study of PRP found the therapy to increase the concentration of various growth factors and upregulate the viability of meniscal cells [8, 14, 39]. Many in vitro studies have demonstrated that the injection of various growth factors could stimulate meniscal tissue repair [40]. By supplying the injury site with a wide range of growth factors, including platelet-derived growth factor, vascular endothelial growth factor, and transforming growth factor beta, PRP promotes cell proliferation, migration, and extracellular collagen matrix formation not only in torn meniscus zones, but also in the entire joint environment [41, 42]. Synoviocytes are affected by platelet releasate, and meniscal cells appear to be induced by PRP and act synergically toward meniscus healing [39]. Consequently, the forest plots demonstrated that the failure rate of meniscus repair treatment in the PRP group improved significantly compared with that in the non-PRP group. Although a recent meta-analysis on the application of PRP in arthroscopic meniscus repair showed no significant difference in the failure rate [43], only three studies on the failure rate were included [19, 30, 33].

The effects of PRP on pain reduction have been previously observed in other studies and several authors have reported the analgesic properties of platelets [44, 45]. Postoperative pain was mainly induced by inflammation [46]. Inflammatory factors (such as interleukin-1*β*, interleukin-6, and tumor necrosis factor-*α*) are released after surgery, which decrease the nociceptor threshold and play an important role in the occurrence of pain [47, 48]. PRP involves the modulation of the meniscal environment by introducing autologous blood products into the targeted tissue, and the growth factors contained in the PRP concentrate can lead to the inhibition of the local inflammatory response and promote chondrogenesis. Moreover, PRP reduces pain by influencing the expression of mediators (such as prostaglandin E2, substance P, dopamine, and 5-hydroxy-tryptamine) [49]. Consequently, our pooled results showed that the postoperative pain VAS scores of the PRP group were significantly lower than those of the control group.

However, the functional outcomes showed no significant difference between the groups regarding KOOS-pain, KOOS-ADL, KOOS-sport/recreation, or KOOS-QoL, except for KOOS-symptom. Neither the IKDC score nor the Lysholm knee scale showed any difference between the non-PRP and PRP groups, which was consistent with previous systematic reviews [22, 23]. Heterogeneity could be the acknowledged significant limitation that resulted from a lack of standardization in PRP dosing and preparation, however, there was no further investigation of this in previous systematic reviews. Therefore, subgroup analyses were firstly performed to evaluate the different PRP preparation systems in our study. Significant correlation of the IKDC score was demonstrated in the PRP subgroup analysis, but no significant correlations were found in the PRFM subgroup analysis.

Consequently, PRP should be recommended instead of PRFM in meniscus repair augmentation. Although positive anabolic effects of PRFM on meniscocytes harvested from the primary culture of a rabbit meniscus were demonstrated [50], PRFM may do the opposite and inhibit meniscus healing. PRFM is a PRP variant whereby a fibrin matrix is formed by activation of the fibrin-clotting cascade, which has to be sutured into the repair site or applied using the fateful rod system, which is a novel device used to deliver platelet-rich fibrin into the joint [20, 32]. As a consequence, the space-occupying effect of fibrin clot in PRFM may result in a gap at the repair site after it dissolves. Additionally, PRFM is known to increase the presence of inflammatory mediators at the repair site [51]. Therefore, fibrovascular scar tissue may have contributed to the initially stronger biomechanical properties of the repair without improving the structural properties with respect to collagen and cartilage organization [52, 53]. Rodeo et al. found that autologous PRFM applied to the tendon-bone interface at the time of surgery did not have a positive effect on the healing tendon-bone interface, tendon vascularity, muscle strength, or shoulder symptoms. Indeed, regression analysis suggested that PRFM may have an inhibitory effect on tendon healing [53], which was consistent with our study that PRFM has been shown to have no benefit in improving functional outcomes.

Moreover, the effect of the leukocyte counts on tissue healing remains greatly debated. Recent research was performed to ascertain whether there was evidence to support the use of LP- or LR-PRP as an adjunct to arthroscopic rotator cuff repair [54]. However, significant differences in platelet concentrations between various commercially produced PRP media remain a confounding variable and make broad generalizations between LR- and LP-PRP based solely on leukocytes impossible [55]. In the present systematic review, there were seven studies utilizing LR-PRP, with two other studies not specifying LR versus LP formulations. Because no studies in this review compared LR- and LP-PRP, no conclusions can be made as to the relative effects. Therefore, we suggest that further studies should be performed to ascertain whether there is evidence to support the use of LP- or LR-PRP as an adjunct to meniscus repair augmentation.

To our knowledge, the present study is the meta-analysis with the largest number of participants to evaluate the use of PRP in meniscus repair treatment. Additional information regarding the heterogeneity issues was obtained by subgroup analyses on the PRP preparation. However, there were still several limitations in our study. First and foremost, the few RCT studies included in this review limit the strength of the conclusions. Second, there is a multitude of confounding factors that may affect the results in our meta-analysis, and this was due to the different types of meniscal injury, repair therapies, and operative technique and the different forms and dosages of PRP applications. Third, because there was an insufficient number of eligible studies, we did not conduct subgroup or meta-regression analyses for the different PRP types, preparations, or applications.

## Conclusions

Although the studies were mostly non-randomized, meniscus repairs augmented with PRP led to significantly lower failure rates and subsequently improved postoperative pain control when compared with repairs without PRP. However, most studies reported no significant differences in patient-reported outcome scores. The findings of our meta-analysis suggest that PRP could be recommended in patients requiring meniscus repair instead of PRFM. In addition, adequately powered prospective randomized trials are needed to further investigate the efficacy of different forms of PRP on meniscus repair treatment because current evidence is limited to small, mostly non-randomized studies and there is a lack of consensus.

## Data Availability

The datasets generated or analyzed during the current study are available from the corresponding author on reasonable request.

## Abbreviations

PRP: Platelet-rich plasma
PRFM: Platelet-rich fibrin matrix
LP: Leukocyte-poor
LR: Leukocyte-rich
RCTs: Randomized controlled trials
PRISMA: Preferred reporting items for systematic reviews and meta-analyses
VAS: Visual analog scale
IKDC: International knee documentation committee
SANE: Single assessment numeric evaluation
KOOS: Knee injury and osteoarthritis outcome score
ADL: Activity of daily living
QoL: Quality of life
WOMAC: Western Ontario and McMaster universities osteoarthritis index
NOS: Newcastle-Ottawa quality assessment scale
ORs: Odds ratios
CI: Confidence interval
MD: Mean difference
MRI: Magnetic resonance imaging
